# Evidence of cAMP involvement in cellobiohydrolase expression and secretion by *Trichoderma reesei* in presence of the inducer sophorose

**DOI:** 10.1186/s12866-015-0536-z

**Published:** 2015-09-30

**Authors:** Karoline Maria Vieira Nogueira, Mariana do Nascimento Costa, Renato Graciano de Paula, Flávia Costa Mendonça-Natividade, Rafael Ricci-Azevedo, Roberto Nascimento Silva

**Affiliations:** Department of Biochemistry and Immunology, Ribeirão Preto Medical School, University of São Paulo, 14049-900 Ribeirão Preto, SP Brazil; Department of Cell Biology and Molecular and Pathogenic Bioagents, Ribeirão Preto Medical School, University of São Paulo, 14049-900 Ribeirão Preto, SP Brazil

**Keywords:** Carbon source, Cellulases, Saprophytic fungus, Secondary messenger, Signaling pathways

## Abstract

**Background:**

The signaling second messenger cyclic AMP (cAMP) regulates many aspects of cellular function in all organisms. Previous studies have suggested a role for cAMP in the regulation of gene expression of cellulolytic enzymes in *Trichoderma reesei* (anamorph of *Hypocrea jecorina*).

**Methods:**

The effects of cAMP in T. reesei were analyzed through both activity and expression of cellulase, intracellular cAMP level measurement, western blotting, indirect immunofluorescence and confocal microscopy.

**Results:**

To elucidate the involvement of cAMP in the cellulase expression, we analyzed the growth of the mutant strain ∆*acy1* and its parental strain QM9414 in the presence of the inducers cellulose, cellobiose, lactose, or sophorose, and the repressor glucose. Our results indicated that cAMP regulates the expression of cellulase in a carbon source-dependent manner. The expression *cel7a,* and *cel6a* genes was higher in the presence of sophorose than in the presence of cellulose, lactose, cellobiose, or glucose. Moreover, intracellular levels of cAMP were up to four times higher in the presence of sophorose compared to other carbon sources. Concomitantly, our immunofluorescence microscopy and western blot data suggest that in the presence of sophorose, cAMP may regulate secretion of cellulolytic enzymes in *T. reesei*.

**Conclusions:**

These results allow us to better understand the role of cAMP and expand our knowledge on the signal transduction pathways involved in the regulation of cellulase expression in *T. reesei.* Finally, our data may help develop new strategies to improve the expression of *cel7a* and *cel6a* genes, and therefore, favor their application in several biotechnology fields.

**Electronic supplementary material:**

The online version of this article (doi:10.1186/s12866-015-0536-z) contains supplementary material, which is available to authorized users.

## Background

*Trichoderma reesei (*anamorph of *Hypocrea jecorina*) is a saprophytic fungus that has an efficient secretion system of cellulolytic proteins involved in the degradation of cell wall polysaccharides of plants. This fungus has an enormous ability to both produce and secrete cellulases. This makes it the most important industrial fungus for the production of these enzymes, which are used for several purposes including the production of bioethanol in the biofuel industry [1]. The cellulolytic system of this fungus consists of at least three different types of enzymes—i.e., exoglucanases (cellobiohydrolases EC 3.2.1.91), endoglucanases (EC 3.2.1.4), and β-glucosidases (EC 3.2.1.21) [2]—that act synergistically and are coordinately expressed in specific growing conditions [3, 4, 5].

In general, fungi belonging to the *Trichoderma* genus have adaptive abilities to colonize different environments. Their survival is ensured through efficient ways to detect cellulose in the environment and secrete various cellulases responsible for the degradation of insoluble substrates, transportation of soluble products via the cytoplasmic membrane, and assimilation of sugars. Furthermore, this fungus has the flexibility to respond to changes in the nutritional composition of the environment, and thus, is able to compete with other microorganisms [6]. Therefore, correct interpretation of environmental stimuli, using the available resources, ensures the success of this fungus in nature [7].

In response to an extracellular signal captured by a receptor, a signaling cascade is initiated, and this signal is transmitted via secondary messengers such as cyclic AMP (cAMP). The cAMP signaling pathway is a cascade that has crucial functions in all organisms and is highly conserved in fungi [7]. In fungi, this messenger is involved in stress response, sporulation, growth, virulence, mycoparasitism, carbon and lipid metabolism as well as other functions in response to extracellular signals [8–12]. It has been suggested that induction of cellulases in *T. reesei* is related to a signaling pathway involving a cAMP-dependent protein kinase, and that, in certain circumstances the presence of this second messenger can cause an overproduction of some cellulases [7, 13]. However, the biosynthesis of these cellulases is not only induced by cellulose, but also by cellobiose, 1,5-δ-lactone celobiono (an analog of cellobiose), lactose and sophorose. On the other hand, readily metabolizable carbon sources such as glucose, fructose, or glycerol repress expression of cellulases [14].

The regulation of transcription of cellulolytic complexes has been extensively studied, and some transcription factors involved in this process have already been identified [15]. However, little is known about how signals are transmitted to these transcription factors. True inductors, receptors, and pathways involved in the transduction of these signals to specific transcription factors have not been identified yet [16]. Many of these factors are induced under the conditions for which they are necessary, and they are degraded once they have done their function. Therefore, it is reasonable to assume that the activity of these transcription factors is also regulated by modifications that occur in response to changes in fungal culture conditions [7].

This study aimed to elucidate the involvement of cAMP in the expression of cellulase genes *cel7a* and *cel6a* in *T. reesei* using different inducers (i.e., cellulose, sophorose, lactose, and cellobiose), and a repressor (i.e., glucose). Our results suggest that cAMP controls the expression of cellobiohydrolases only in the presence of the inducer sophorose and is potentially involved in the secretion process of cellulases.

## Methods

### Strains and growth conditions

*T. reesei* QM9414 (ATCC 26921) and mutant ∆*acy1* [17] strains were obtained from the Institute of Molecular Biotechnology, Vienna, Austria. Strains were maintained in MEX medium [malt extract 3 % (w/v) and agar-agar 2 % (w/v)] at 4 °C. QM9414 and ∆*acy1* were grown in MEX medium at 28 °C for seven to 10 days to complete sporulation. For gene expression assays and cellulase activity measurements in QM9414, a suspension containing approximately 10^8^ spores/mL was inoculated in 200 mL Mandels-Andreotti medium [18] containing 1 % (w/v) cellulose (Avicel, Sigma, St. Louis, Missouri, EUA), or 1 % (w/v) lactose (Sigma, St. Louis, Missouri, EUA), or 1 % (w/v) cellobiose (Serva, Heidelberg, Germany), or 2 % (w/v) glucose (Sigma, St. Louis, Missouri, EUA), or in 20 mL of the same medium containing 1 mM sophorose (Serva, Heidelberg, Germany) as the sole carbon source. For gene expression assays in ∆*acy1*, a suspension containing approximately 10^8^ spores/mL was inoculated in 200 mL Mandels-Andreotti medium containing 1 % (w/v) cellulose (Avicel), or in 20 mL of the same medium containing 1 mM sophorose as the sole carbon source, with or without 1 mM dibutyryl-cAMP (dbcAMP - Sigma, St. Louis, Missouri, EUA). Cultures were incubated on an orbital shaker (200 rpm) at 28 °C for 24, 48, and 72 h using cellulose, lactose, or cellobiose; for 24 and 48 h using glucose; and 2, 4 and 6 h, or 6, 12 and 18 h using sophorose. For the latter, the mycelium was previously grown in 1 % (w/v) glycerol (Sigma, St. Louis, Missouri, EUA) for 24 h. After incubation, the mycelia were washed with Mandels-Andreotti medium without peptone, and transferred to 20 mL of Mandels-Andreotti medium without peptone containing 1 mM sophorose. All experiments were performed in three biological replicates. The resulting mycelia and supernatants were collected by filtration, frozen and stored at −80 °C until RNA and protein extraction, as well as cellulose activity measurements were performed.

### Determination of cellulase activity

Determination of cellulase activity was performed using Cellulose Azure® (Sigma, St. Louis, Missouri, EUA) as the substrate. This methodology involves the release of a blue color when cellulases are present. In these experiments, the reaction mixture consisted of Cellulose Azure, 100 mM sodium citrate buffer pH 5.0, and samples from culture supernatants. Reactions were performed at 55 °C for 30 min. After this, the alcohol precipitation reagent (APR) was added according to the manufacturer instructions, and samples were centrifuged. Supernatant was collected, and samples were measured using a spectrophotometer (Spectrophotometer xMark™ Micro, Bio-Rad, San Francisco, CA, USA) at 575 nm. A calibration curve was created using varying concentrations of the substrate incubated at 50 °C for 4 h in 100 mM sodium citrate buffer pH 5.0 and 15 U/mL cellulase purified from *Trichoderma reesei* in the Microbiology Laboratory of Cell Biology at the Biology Department (Faculty of Philosophy, Sciences, and Letters of Ribeirao Preto, University of Sao Paulo, Brazil). Activity was expressed as the ratio between change in growth factor absorbance and the absorbance obtained in the standard curve, per min, and per amount of enzyme added to the reaction.

### RNA extraction

Total RNA was extracted from mycelia of each sample using TRIzol® RNA kit (Invitrogen Life Technologies, Carlsbad, CA, USA), according to the manufacturer instructions. RNA concentration was determined using spectrophotometric optical density at 260/280 nm, and RNA integrity was verified by electrophoresis in 1 % agarose gels.

### Quantitative real time-PCR analysis

Approximately 1 μg RNA was treated with DNAseI (Fermentas, Waltham, Massachusetts, USA) and reverse-transcribed to cDNA using the First Strand cDNA kit Maxima™ Synthesis (Thermo Scientific, Waltham, Massachusetts, USA) according to manufacturer instructions. cDNA was diluted 1:50 for real-time PCR analysis in a Bio-Rad CFX96™ System (Bio-Rad, San Francisco, CA, USA), using SsoFast™ EvaGreen® Supermix (Bio-Rad, San Francisco, CA, USA) for signal detection, in accordance with the manufacturer instructions. Primers used in this study are listed in Table 1. The gene encoding actin was used as a constitutive expression control [19]. The following amplification reaction was used: 95 °C for 10 min followed by 39 cycles at 95 °C for 10 s, 60 °C for 30 s followed by a dissociation curve at 60 to 95 °C with an increment of 0.5 °C every 10 s. Gene expression values were calculated according to the 2^-ΔΔCT^ method, using the QM9414 strain grown in glucose as the reference sample [19]. Data analysis was performed using GraphPad Prism v5.1 software.

**Table 1:** Primers used in this study

No.	Code	Protein ID	5’→3’
1	Cel6a F	72567	ACA AGA ATG CAT CGT CTC CG
2	Cel6a R		TGT TCC ACC CGT TGT AGT TG
3	Cel7a F	123989	CCG AGC TTG GTA GTT ACT CTG
4	Cel7a R		GGT AGC CTT CTT GAC TGA GT
5	Actin F	44504	TGA GAG CGG TGG TAT CCA CG
6	Actin R		GGT ACC ACC AGA CAT GAC AAT GTT

### Extraction and measurement of cAMP

After fungal growth in Mandels-Andreotti medium, mycelia were collected and frozen in liquid nitrogen. For measurement of cAMP, mycelia were macerated, and samples were transferred to polypropylene tubes and weighed. Then, 10 volumes of 0.1 M HCl were added, and the tubes were centrifuged at 6,000 × *g* for 10 min at 4 °C. The supernatant was used directly for measurement of cAMP levels using the “Direct cAMP Enzyme Immunoassay” kit (Sigma, St. Louis, Missouri, EUA) according to the manufacturer instructions. The content of intracellular cAMP was relative to protein concentration in the same sample.

### Protein extraction and western blot

Secreted proteins from QM9414 and ∆*acy1* strains were precipitated using 10 % tricarboxylic acid (TCA) in acetone, and allowed to stand at −20 °C overnight. Samples were centrifuged at 10,000 × *g* for 10 min at 4 °C, and the supernatant was removed. β-mercaptoethanol (0.07 %) in acetone was added, and samples were centrifuged at 10,000 × *g* for 10 min at 4 °C. This process was repeated three times, discarding the supernatant after each centrifugation. Then, samples were resuspended in buffer (25 mM Tris pH 6.8, 12.5 % SDS, 50 % glycerol and 0.05 % bromophenol blue) and heated for 5 min at 100 °C. For intracellular protein extraction [20], mycelia grown in sophorose were used as the source [21]. Total protein concentration was determined according to the Bradford method using the Coomassie Plus Protein Assay Reagent (Thermo Scientific, Waltham, Massachusetts, USA) at 595 nm, in a spectrophotometer (Spectrophotometer xMark™ Micro, Bio-Rad). Subsequently, 12 μg of protein samples (secreted and intracellular proteins) were subjected to electrophoresis on 10 % polyacrylamide gels. After electrophoresis, proteins were transferred to nitrocellulose membranes (GE Healthcare) for 40 min using a humid system (Trans-Aid ™ Blot®Turbo transfer system, Bio-Rad) with transfer buffer (25 mM Tris, 197 mM glycine, 20 % methanol). Membranes were blocked for 1 h at room temperature in tris-buffered saline (TBS) containing 0.05 % Tween (TBS-Tween) and 5 % skimmed milk. Membranes were then incubated overnight at 4 °C with a rabbit polyclonal anti-*Trichoderma viride* cellulase (CEL7A) antibody (MyBioSource) diluted 1:1000. Membranes were then washed three times 10 min each with TBS-Tween and incubated for 1 h with the corresponding peroxidase-conjugated anti-rabbit secondary antibody (Invitrogen). Membranes were again washed three times 10 min each with TBS-Tween and developed using Enhanced Chemiluminescence reagent (ECL, GE Healthcare) according to the manufacturer instructions. Finally, ECL membranes were photographed using ChemiDoc™ XRS+ (BioRad) photo-documentation system.

### Indirect immunofluorescence and confocal microscopy

Aliquots withdrawn from Δ*acy1* at 6, 12, and 18 h during culture were centrifuged at 6,000 × *g* and washed with 0.1 M phosphate buffered saline (PBS) at pH 7.4. After washing, mycelia were immersed in 2 % paraformaldehyde in PBS at room temperature and embedded in optimal cutting temperature (OCT) compound (Tissue Tek, Sakura Finetec, Torrence CA) in a suitable tissue mold for freezing. Subsequently the inclusion, mycelia were sectioned on a cryostat (10 μm thick), and sections were mounted on silanized slides kept under vacuum for 12 h to affix the sections. Slides were then stored at −20 °C. For confocal microscopy, slides were washed with PBS and incubated with a 3 % bovine serum albumin (BSA) solution for 1 h at room temperature to block non-specific binding. Then, slides were incubated for 1 h at room temperature with a rabbit polyclonal anti-*Trichoderma viride* cellulase (CEL7A) antibody (MyBioSource) diluted 1:1000 in 3 % BSA solution in PBS. Slides were then incubated with an Alexa Fluor 488-conjugated secondary antibody (Molecular Probes, Life Technologies) diluted 1:5000 in PBS with 3 % BSA at room temperature. Slides were washed with PBS between steps and images were acquired using a Leica TCS SP5 confocal microscope.

### Analytical methods

Biomass was determined by gravimetric analysis for glycerol culture. After 24 h of culture, mycelia was filtrated on filter paper and incubated at 70 °C for 3 h and then weighed. For cellulose culture, biomass was indirectly measured by the amount of intracellular protein quantified by the Quick Start Bradford protein assay kit (Bio-Rad) with bovine serum albumin (BSA) as a standard.

## Results

### Analysis of the expression of the cellobiohydrolases cel7a and cel6a and cellulase activity using different carbon sources

**Fig. 1:**
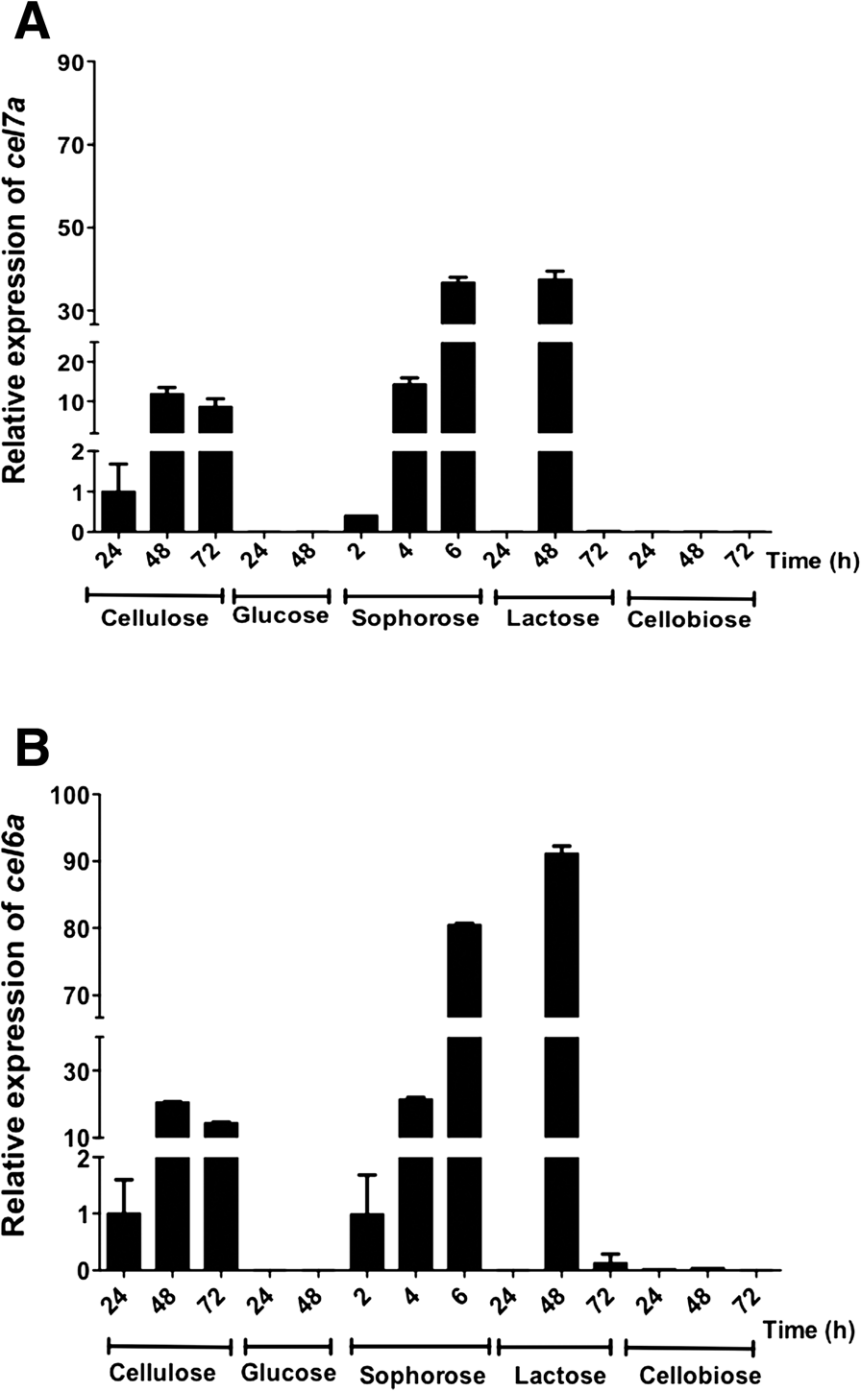
Gene expression levels of *cel7a* (**a**) and *cel6a* (**b**). *T. reesei* QM9414 strain was grown in cellulose, lactose, or cellobiose for 24, 48, and 72 h, in glucose for 24, and 48 h, and in sophorose for 2, 4, and 6 h. The absolute expression of the genes was calculated using actin gene as an endogenous control

To guarantee comparisons between strains, we first examined the growth pattern of strains on cellulose and glycerol. No significant difference in growth between ∆*acy1 mutant strain* and the parental QM9414 as in cellulose as in glycerol was observed [see Additional file 1]. Expression of the two most expressed cellulase genes in *T. reesei* (*cel7a* and *cel6a*) was analyzed after growing the QM9414 strain in different carbon sources, as described in the Methods section. As shown in Fig. 1, *cel7a* and *cel6a* expression increased when the fungus was grown in cellulose, sophorose, or lactose. Using cellulose, *cel7a* expression increased 10-fold at 48 and 72 h (Fig. 1a). Regarding sophorose and lactose, the highest *cel7a* expression (30-fold) was observed after 6 and 48 h, respectively. The expression profile of cellobiohydrolase *cel6a* was similar to that of *cel7a,* with the highest expression levels in sophorose at 6 h (80-fold) and lactose at 48 h (90-fold) (Fig. 1b). As expected, neither *cel7a* nor *cel6a* showed detectable expression levels in glucose. Interestingly, no expression of *cel7a* and *cel6a* was observed in cellobiose.

**Fig. 2:**
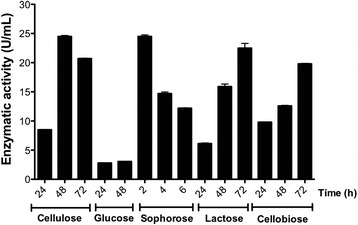
Total cellulolytic activity in different carbon sources. *T. reesei* QM9414 strain was grown in cellulose, lactose, or cellobiose for 24, 48, and 72 h, in glucose for 24, and 48 h, and in sophorose for 2, 4, and 6 h. Activity was measured using the azure-Cellulose method and is expressed as the ratio between growth factor and the absorbance obtained in the standard curve equation, per minute, and per amount of enzyme added to the reaction

Similar to qPCR-RT results, the QM9414 strain exhibited higher cellulolytic activity in cultures with cellulose, sophorose, and lactose (Fig. 2). However, different to what was observed in the expression analysis, which showed an increase of *cel7a* and *cel6a* expression after 6 h, cellulolytic activity in sophorose increased mainly at 2 h in culture (24.5 U/mL). A similar pattern was observed growing QM9414 in lactose, where cellulolytic activity reached high levels at 72 h (22.5 U/mL). Cellulolytic activity in cellulose was higher at 48 and 72 h (24.5 U/mL and 20.7 U/mL, respectively). On the contrary, hydrolytic activity in glucose was lower than in other carbon sources, being 6, 5, and 4.8 times lower than in cellulose, sophorose, lactose and cellobiose, respectively. Interestingly, our results showed no correlation between the expression profile and cellulolytic activity in cellobiose. The cellulolytic activity showed a steady increase from 24 h and a maximum at 72 h (19.8 U/mL) (Fig. 2). This result may be explained from the fact that the azure-Cellulose® method detects total cellulolytic activity without distinguishing specific cellulases.

### Intracellular cAMP levels are regulated according to the carbon source

We found that cellobiohydrolase gene expression and cellulolytic activity profile varied depending on the carbon source used in the experiments of induction. Previous studies have suggested that intracellular levels of cAMP may regulate both expression of cellulase genes and activity of the enzyme. To address this, we measured intracellular levels of cAMP in mycelia from the QM9414 strain grown in cellulose, sophorose, lactose, cellobiose, and glucose.

**Fig. 3:**
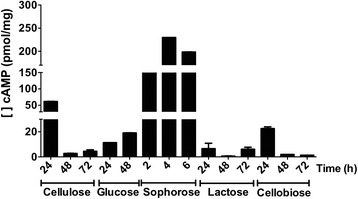
Intracellular cAMP content. *T. reesei* QM9414 strain was grown in cellulose, lactose, or cellobiose for 24, 48, and 72 h, in glucose for 24, and 48 h, and in sophorose for 2, 4, and 6 h

Our results showed that intracellular levels of cAMP were modulated in a carbon source-dependent manner. As shown in Fig. 3, cAMP concentration (229.8 pmol/mg) at 4 h was at least 4 times higher in the presence of sophorose than in the presence of other carbon sources. In cellulose, the highest cAMP concentration was quantified after 24 h cultivation (61.7 pmol/mg). This value was 3 times higher than the maximum levels found in glucose (19.13 pmol/mg) at 48 h and 2.5 times higher than with cellobiose (22.5 pmol/mg) at 24 h (Fig. 3). Concomitantly, lower levels of cAMP were detected in lactose and cellulose at 48 and 72 h, and cellobiose at 48 and 72 h. Altogether, our findings showed that in a similar manner to cellobiohydrolase expression and cellulolytic activity, intracellular levels of cAMP are also dependent on the inducer. This suggests a regulatory role of cAMP in the control of cellulase expression only in the presence of sophorose, since expression levels of *cel7a* and *cel6a* were increased in both lactose and cellulose. On the other hand, intracellular concentration of cAMP did not exhibit the same profile regarding carbon sources.

### Analysis cel7a and cel6a expression in the ∆acy1 mutant strain

The expression profile of the two most abundant cellobiohydrolases in the parental lineage QM9414 was directly regulated in response to four different inducers. Here we showed that the increased expression of *cel7a* and *cel6a* correlates with high intracellular levels of cAMP in the presence of sophorose. Then, we wanted to assess how cAMP regulates cellulase expression in *T. reesei*, and whether the effects of this secondary messenger are determined by the carbon source. To assess this, we used the ∆*acy1* mutant strain, which features a deletion in the adenylate cyclase gene, and dbcAMP (dibutyryl-cAMP) in the induction medium. Growth of the ∆*acy1* mutant strain was assessed in cellulose and sophorose at 72 and 6 h, respectively, which were the time points and carbon sources that showed the highest expression of cellobiohydrolases.

**Fig. 4:**
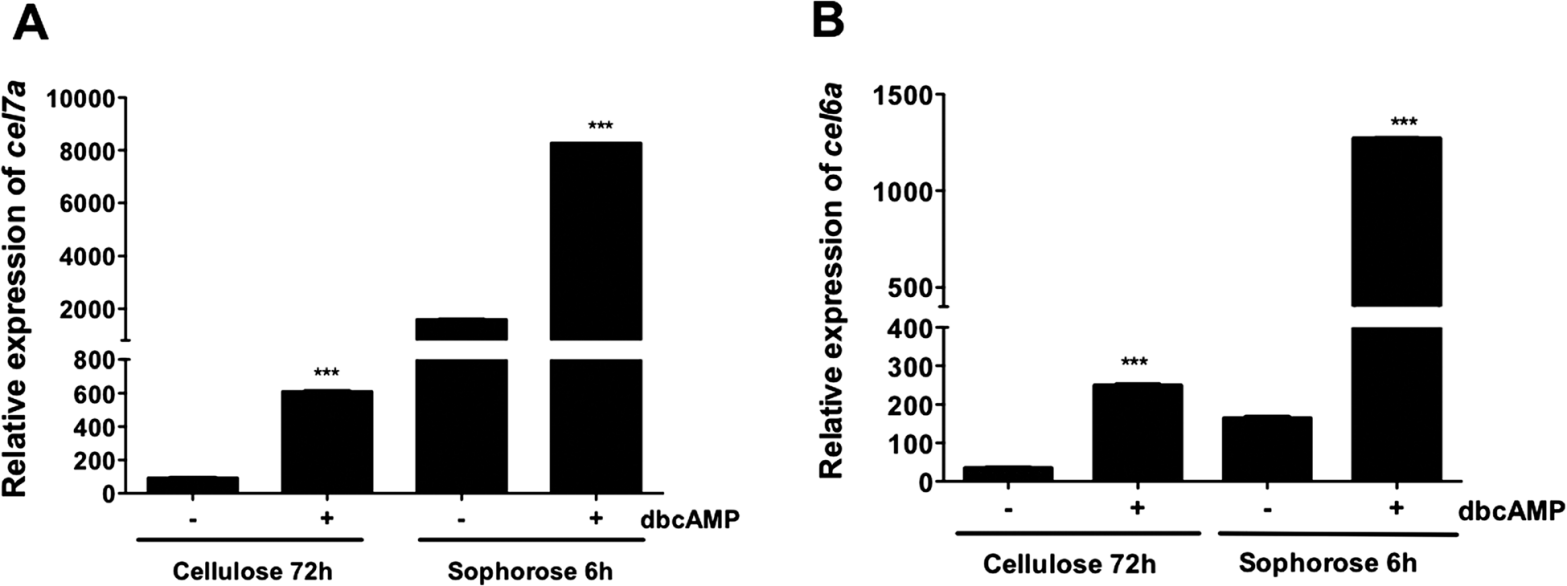
Gene expression levels of *cel7a* (**a**) and *cel6a* (**b**). *Δacy1* mutant strain was grown in cellulose for 72 h, and in sophorose for 6 h. The absolute expression is shown, with the actin gene used as an endogenous control. Gene expression was analyzed with or without addition of 1 mM dibutyryl-cAMP (dbcAMP)

Figure 4 shows that in the presence of sophorose and dbcAMP, expression of the cellobiohydrolases *cel7a* (Fig. 4a) and *cel6a* (Fig. 4b) was significantly increased relative to cellulose, either in the presence or absence of dbcAMP. Interestingly, dbcAMP effects were mainly evident in the presence of the most potent inducer of cellulase expression (i.e., sophorose) than in cellulose at the same concentration. As a result of the abolition of adenylate cyclase expression in the ∆*acy1* mutant strain, the only source of cAMP in the induction medium was dbcAMP. Therefore, supplementation with exogenous cAMP enhanced the expression of cellobiohydrolases in the ∆*acy1* strain, suggesting an essential effect of cAMP in the regulation of cellulase gene expression in *T. reesei.*

### Detection of total cellulase in mycelia and secretome of ∆acy1 and QM9414

The cAMP signal transduction pathway controls a wide variety of processes in fungi. In the ∆*acy1* mutant strain processes such as carbon metabolism, conidiation, mating, phototropism, and synthesis and secretion of proteins may be disturbed because of adenylate cyclase deletion. Using western blot and indirect immunofluorescence microscopy approaches we assessed cellulase content in the mycelia (intracellular protein) and in the secretome of QM9414 and ∆*acy1* strains.

**Fig. 5:**
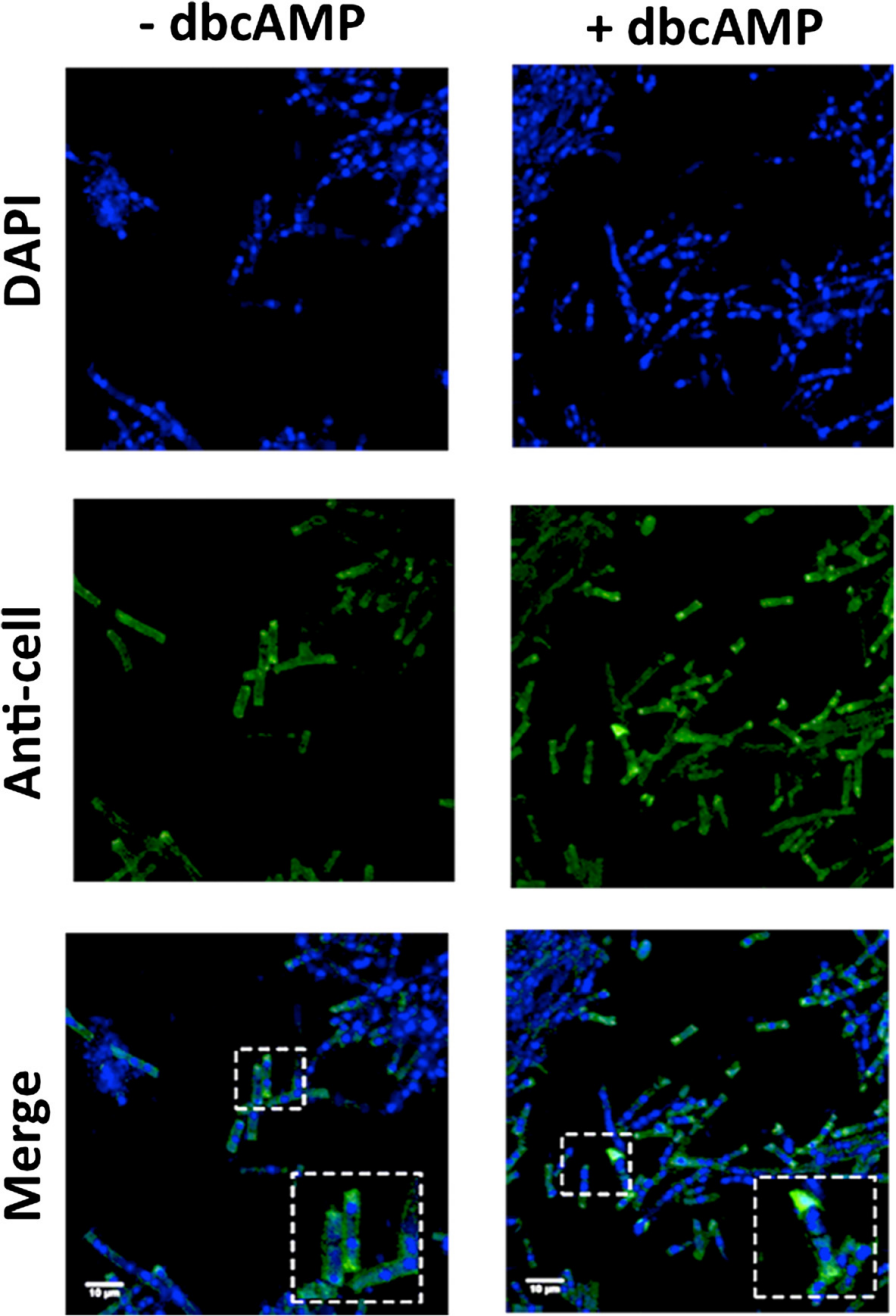
Cellulase detection using indirect immunofluorescence in the *Δacy1* mutant strain. The fungus was grown in sophorose for 6 h in the presence or absence of dibutyryl-cAMP (dbcAMP). To detect cellulase in the mycelia we used a rabbit polyclonal anti-*Trichoderma viride* cellulose (CEL7A) antibody diluted 1:1000, and an Alexa Fluor 488-conjgated secondary antibody diluted 1:5000. Furthermore, the fluorochrome DAPI was used to detect cell nuclei. Images were acquired on a Leica TCS SP5 confocal microscope. Lens 40x, and zoom 3.8

Our immunofluorescence results revealed that dbcAMP strongly influenced cellulase expression in the ∆*acy1* mutant strain (Fig. 5). Addition of dbcAMP promoted an increase in cellulase expression at 6 h in induction medium with sophorose. Similar results were also observed at 12 and 18 h (Data not shown), with the same increase in cellulase expression. We observed an accumulation of cellulases in the hyphal tip (Fig. 5, dashed squares), when dbcAMP was added. These results, together with the qRT-PCR and activity analyses, suggest that dbcAMP directly controls expression and secretion of cellulase in *T. reesei*.

**Fig. 6:**
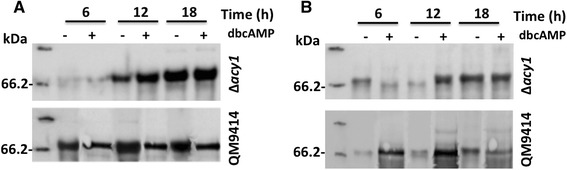
Analysis of cellulase expression in *Δacy1* and QM9414 strains. Fungi were grown in sophorose for 6, 12, and 18 h in the presence or absence of dibutyryl-cAMP (dbcAMP). **a** Expression of CEL7A in the secretome of the *Δacy1* mutant strain (upper panel) and QM9414 parental strain (lower panel) in the presence or absence of dbcAMP. **b** Expression of CEL7A in the mycelium of the *Δacy1* mutant strain (upper panel) and QM9414 parental strain (lower panel) in the presence or absence of dbcAMP

We found no correlation between expression level of cellobiohydrolases and activity in at 6 h of incubation in the presence of sophorose. On the other hand, the immunofluorescence analysis showed that at 6 h in the presence of dbcAMP there are increased of cellulase expression. So, we asked if occurs a retention of cellulases in the mycelium. Our *western blotting* results showed a detection of an approximately band of 59–68 kDa (relative to CEL7A), and we observed a lower content of CEL7A in the secretome of ∆*acy1* strain in the absence of dbcAMP (Fig. 6a, upper panel). The addition of dbcAMPC increased the content of CEL7A in the secretome. The differences were more evident at 12 h and 18 h, being the higher expression of CEL7A reached always in the presence of dbcAMP. The detection of CEL7A in the mycelium of ∆*acy1* (Fig. 6b, upper panel) revealed an elevated content of this cellulase at 6 h in the absence of dbcAMP, justifying the lower detection in the secretome. In the same way, the addition of dbcAMP at 6 h induced a high secretion of CEL7A as demonstrated by the decreased of protein in the mycelium (Fig. 6b, upper panel). In addition, we observed that the content of CEL7A in the mycelium at 12 h and 18 h were higher than at 6 h, but the content of protein in the secretome remain increased at 12 h and 18 h relative to mycelium. In the ∆*acy1* strain the expression of cellulase was higher after 6 h of cultivation, being the content of CEL7A maximum at 18 h. Oppositely, in the secretome of QM9414, the maximum content of CEL7A was detectable at 12 h in the absence of dbcAMP (Fig. 6a, lower panel). Moreover, we demonstrated a lower content of cellulase in the mycelium of QM9414 at 6 h, 12 h and 18 h compare to *∆acy1* mutant strain (Fig. 6b). So, the analysis of secretome and mycelium protein content revealed that addition of dbcAMP alters the pattern of CEL7A secretion in *T. reesei*. The content of CEL7A in the presence of dbcAMP had no change in the secretome, but comparing the protein content in the mycelium in the absence and in the presence of dbcAMP, we observed the accumulation of protein in the mycelium after addition of dbcAMP mainly at 6 h and 12 h (Fig. 6b, lower panel). Our findings suggest that cAMP may regulate the expression, and secretion of cellobiohydrolases in *T. reesei* in the presence of the inducer sophorose.

## Discussion

In *T. reesei*, it is well established that expression of cellulases is carbon source-dependent [12]. Our group has described the influence of different carbon sources in the regulation of cellulase gene expression [22, 4]. Although various studies have discussed that cellulase genes are dependent on induction, little is known about the nature of the inducer, and the signaling pathways controlling cellulase gene expression in this fungus [16].

In *T. reesei* the only well characterized signal transduction cascades are: a light-modulated cellulase production (G protein-mediated, Gna1 and Gna3), a PAS/LOV domain protein ENVOY and cAMP-dependent protein kinase A signaling [7, 17, 18, 23], regulation of sexual development [24], and cellulose- and cAMP-independent modulation of cellulase production mediated by Ras GTPase [25]. Recently, Wang et al. [26, 27] described the role of MAPK signaling in the regulation of both expression and activity of cellulases in *T. reesei*.

The cyclic AMP (cAMP) pathway is a central signaling cascade with crucial functions in all organisms. In *Saccharomyces cerevisiae,* nutrient sensing and pseudohyphal differentiation in response to nitrogen-limiting conditions is controlled by this pathway [28–30]. In the fission yeast, *Schizosaccharomyces pombe*, cAMP mediates the effect of glucose and gluconeogenesis in spore germination, and regulates mating in response to either glucose or nitrogen deprivation [31, 32]. In filamentous fungi such as *Neurospora crassa* and *Aspergillus* species, cAMP controls hyphal growth polarity and morphogenesis, conidiation, and spore germination [33–36]. Furthermore, as a secondary messenger, cAMP is involved in stress response, carbon and lipid metabolism, sporulation, development, virulence, mating, mycoparasitism, and other responses to extracellular signals [8–12, 37].

Previous studies in *T. reesei* have suggested that cAMP signaling regulates cellulase gene expression [13]. Sestak and Farkas (1993) [13] showed that addition of cAMP can double the efficacy of sophorose in the induction of endoglucanase formation. In another filamentous fungus, *Cryptococcus albidus*, cAMP was shown to be important for xylanase production [38]. According to Tisch and Schmoll [7], the most important role of cAMP is to activate cAMP-dependent protein kinase A (PKA), which in turn initiates a phosphorylation cascade and activates/inactivates further target genes. Schuster et al. (2012) [17] showed that both adenylate cyclase (ACY1) and PKA (catalytic subunit, PKAC1) were important factors in cellulase gene expression in *T. reesei*. However, the mechanisms related to the impact on cellulase gene expression remain unclear.

In the present study, we showed that the effect of cAMP in the regulation of cellulase gene expression in *T. reesei* was carbon source-dependent and that the role of this secondary messenger is more evident in the presence of sophorose. This sugar is formed by transglycosylation of cellobiose during cellulose hydrolysis [39], and it is thought to be the natural inducer of cellulase formation, being its presence in culture fluids of *T. reesei* commonly described [40–42]. Moreover, this disaccharide has a dual role in *T. reesei* in β-glucosidase repression and cellulase induction [16]. The efficiency of sophorose and other disaccharides such as lactose in the induction of cellulase has also been observed in *Trichoderma viride*, *Pseudomonas fluorescens var. cellulosa*, *Acremonium cellulolyticus, Penicillium echinulatum* [41, 43–45]. Interestingly, our results showed a low expression of cellulase genes in the presence of cellobiose. This may be explained by the fast hydrolysis of this sugar by β-glucosidase activity, which converts cellobiose to glucose, a carbon source repressor of cellulase synthesis [46, 47]. Altogether, our results suggest an intriguing mechanism by which the fungus identifies the available carbon source in the culture medium and controls the full transcription machinery of genes responsible for carbon source metabolism. In addition, our results highlight the role of cAMP as a sophorose sensor.

Our qRT-PCR results showed that expression of cellobiohydrolases *cel7a* and *cel6a* increased in the presence of cellulose, sophorose, and lactose. However, in the presence of sophorose, addition of dbcAMP induced an increase of 5- and 7.8-fold in the expression of *cel7a* and *cel6a*, respectively. As observed, the expression of *cel6a* was more sensitive to intracellular levels of cAMP than *cel7a*. Interestingly, the high expression levels of *cel7a* and *cel6a* correlated with high intracellular cAMP levels. The influence of cAMP in the regulation of cellulase expression has been shown in important fungi such as *Trichoderma*, *Penicillium,* and *Aspergillus* spp., which have biotechnological interest [41, 45]. Furthermore, Hu et al. [48], in a study in *Penicillium decumbens*, showed the effect of PGA3, a group III G-protein α subunit, on the expression of amylases and cellulases. Deletion of *pga3* resulted in impaired amylase production, and significantly decreased transcription of the major amylase gene *amy15A.* Moreover, supplementation with exogenous cAMP or its analog dbcAMP restored amylase production in the Δ*pga3* strain, suggesting an essential role for PGA3 in amylase synthesis by controlling cAMP levels. Conversely, transcription of the cellulase gene *cel7A-2* increased in the Δ*pga3* strain, although cellulase activity in the medium was not affected. Dong et al. [49] showed that exogenous cAMP could increase cellulase synthesis under derepression conditions. Nevertheless, cAMP has an ambiguous role in the regulation of cellulase expression because at lower concentrations increases cellulolytic activity, while high levels of cAMP repress cellulase synthesis. Interestingly, Herrera-Herrera et al. [50] showed that in *Cellulomonas flavigena*, an actinobacteria with special interest for its ability to degrade cellulose and hemicellulose, addition of exogenous cAMP in repressor culture medium decreased catabolite repression, while supplementation enhanced cellobiohydrolase production. Similarly, Rizzati et al. [51] showed in *Aspergillus phoenicis* that repression of xylanase expression by glucose was partially reversed by addition of cAMP or dbcAMP.

In our experiments using cellulose, we showed that expression of *cel7a* and *cel6a* in the ∆*acy1* strain were 6- and 7.3-fold higher with addition of dbcAMP. This increase may be explained because degradation of cellulose releases inducer disaccharides such as sophorose, which in turn may active the expression of cellulolytic enzymes. This result corroborates our hypothesis that cAMP affects the expression of cellobiohydrolase, and that this regulation seems to be dependent on the type of carbon source, since changes in transcription of related genes were observed in both cellulose and sophorose, using the same concentration of dbcAMP. Hu et al. [48] described a different regulation in *P. decumbens*, in which expression of amylases and cellulases seemed to be independent of the carbon source. Taken together, our results suggest that the signaling pathway in response to sophorose involves a cAMP-dependent protein kinase that may control the expression of cellulolytic enzymes in the presence of easily metabolizable disaccharides, and that is also involved in the early stage of carbon sensing.

Our results revealed that the ∆*acy1* mutant strain showed a high expression of cellobiohydrolases in the presence of both cellulose and sophorose. As discussed above, this expression increased with supplementation of exogenous cAMP. This finding suggests that cAMP is not essential for cellulase expression, and that alternative pathways may interact with cAMP-dependent signaling to control expression of cellulolytic enzymes. In this regard, Schmoll et al. [18] showed that in *T. reesei* intracellular cAMP levels were positively correlated with cellulase expression in the presence of light. Furthermore, sensing of environmental signals mediated by G-protein coupled receptors (GNA1 and GNA3) modulates considerably cellulase transcript levels, and the extent of this adjustment is dependent on light status. The critical light regulator ENVOY in *T. reesei* [52], is a small protein that contains a single PAS/LOV domain. The expression of the *env1* gene is very low in darkness, but upon illumination the abundance of its transcript increases up to 500-fold. Recently, Tisch et al. [7] reported that ENVOY is involved in signal transduction via G-proteins, acting positively in the feedback of *gna1* and in the cAMP/protein kinase A pathway, controlling the function of the corresponding phosphodiesterase, although the mechanism is still unclear. In *T. atroviride*, a GPCR (GPR-1) that activates heterotrimeric G-proteins senses different carbon sources, and the Gα proteins (*tga1* or *tga3)* can activate adenylate cyclase that in turn may control intracellular levels of cAMP [53]. Thus, our results obtained in the ∆*acy1* strain provide evidence of the existence of a cAMP-independent pathway that controls cellulase expression, and may involve a receptor that senses different carbon sources; the effects of this sensing are more evident in the presence of disaccharides such as sophorose. These results are also in agreement with other studies [13, 54, 55], which showed that cAMP may be an important signal for the regulation of cellulase formation, but not the only one.

The effect of cAMP on the regulation of cellulase expression in *T. reesei* was also revealed in our comparative analysis of immunofluorescence and western blot. Immunofluorescence data clearly showed that supplementation with exogenous cAMP controls expression of *cel7a* in sophorose induction. In addition, we observed that in the *∆acy1* mutant strain there is a delay in the secretion of cellobiohydrolase CEL7A compared to the parental QM9414. Kinetic studies have shown that secretion is not faster in *T. reesei* than in other species. However, a good secretion capability depends on the capacity of the secretion machinery [56]. Altogether, our results suggest an involvement of intracellular cAMP levels in the regulation of cellulase secretion.

Filamentous fungi such as *T. reesei* and *A. niger*, produce large amounts of extracellular cellulolytic enzymes, while some strains mostly produce them in a multi-enzyme complex called cellulosome, which is associated with the degrading cell wall [57–61]. Usually, a typical secretory pathway in a cell is composed of at least two components, endoplasmic reticulum and Golgi apparatus, and two endomembrane systems, one for incoming, and another for outgoing traffic [62]. On the other hand, secretion of cellulase suggests the existence of three different mechanisms based on their subcellular location: a specific secretory pathway independent of cellulose, a secretory pathway, which is induced by cellulose, and a process that occurs irrespective of the carbon source [63].

Cellulase secretion needs to be induced, and this process involves the synthesis of new proteins for constructing secretory pathways. In *A. niger*, de Oliveira et al. [64] described the induction of 254 different predicted proteins related to the secretory pathway. Interestingly, the induction of proteins seemed to be carbon source-dependent. Similarly, studies in *Clostridium thermocellum* showed that endoglucanase activity was regulated by carbon source [65–68]. In *A. nidulans*, non-essential protein kinases and phosphatases were involved in the sensing of carbon and/or energetic status, and in the regulation of hydrolytic enzyme production [69]. As discussed above, cAMP controls a wide range of processes in the cell, including activity of kinase proteins. The *∆acy1* strain exhibited an increase in the cellobiohydrolase expression. Interestingly, upregulation of *cel7a* and *cel6a* was more evident and the intracellular cAMP content was higher in the presence of sophorose than in the presence of other carbon sources tested. Furthermore, secretion of CEL7A was altered compared to the QM9414 parental strain. These results suggest that sophorose may be sensed by a different signaling pathway in the *∆acy1* mutant strain, and that mutation of the *acy1* gene may alter the pattern of cellulases secretion in this strain. However, additional studies will be needed to identify potential kinase proteins involved in this process in *T. reesei*.

## Conclusions

The present study contributes to a better understanding of the role of cAMP signaling pathway in the regulation of cellulase expression in *T. reesei*. Our results showed that cAMP effects are carbon source-dependent, with regulation of cellobiohydrolases more evidently affected in the presence of sophorose. Interestingly, cellulase secretion was altered in the *∆acy1* mutant strain, in which cAMP synthesis is disrupted. Moreover, our study is the first report discussing a potential role for cAMP in the regulation of cellulase secretion in *T. reesei*. These results suggest that cAMP is an important signaling pathway involved in cellulase expression in *T. reesei*, but only in the presence of sophorose. These findings contribute to the understanding of the molecular mechanisms involved in the regulation of the processes of cellulolytic enzyme synthesis and secretion.

## Additional file

Additional file 1: Figure S1. Growth profiles of *T. reesei* QM9414 and Δ*acy1* strains in cellulose (A) and glycerol (B) as carbon source. Error bars are represented from three biological replicates. No significant difference was showed in growth between ∆acy1 mutant strain and the parental QM9414 (PDF 117 kb)

## Abbreviations

ACY1: Adenylate cyclase
BSA: Bovine serum albumin
cAMP: Cyclic AMP
APR: Alcohol precipitation reagent
dbcAMP: Dibutyryl-cAMP
ECL: Enhanced chemiluminescence
MEX: malt extract
PBS: Phosphate buffered saline
PKA: Protein kinase A
qRT-PCR: Quantitative real time-PCR
SDS: Sodium dodecyl sulfate
TCA: Trichloroacetic acid
TTBS: Tris-buffered saline
