# Supplementary material for: Evidence of cAMP involvement in cellobiohydrolase expression and secretion by Trichoderma reesei in presence of the inducer sophorose
Source: BMC Microbiol. 2015 Sep 30;15:195. doi: 10.1186/s12866-015-0536-z (PMC4590280; doi:10.1186/s12866-015-0536-z)
Supplement: Additional file 1: Figure S1. — Growth profiles of T. reesei QM9414 and Δacy1 strains in cellulose (A) and glycerol (B) as carbon source. Error bars are represented from three biological replicates. No significant difference was showed in growth between ∆acy1 mutant strain and the parental QM9414 (PDF 117 kb) [file 12866_2015_536_MOESM1_ESM.pdf]

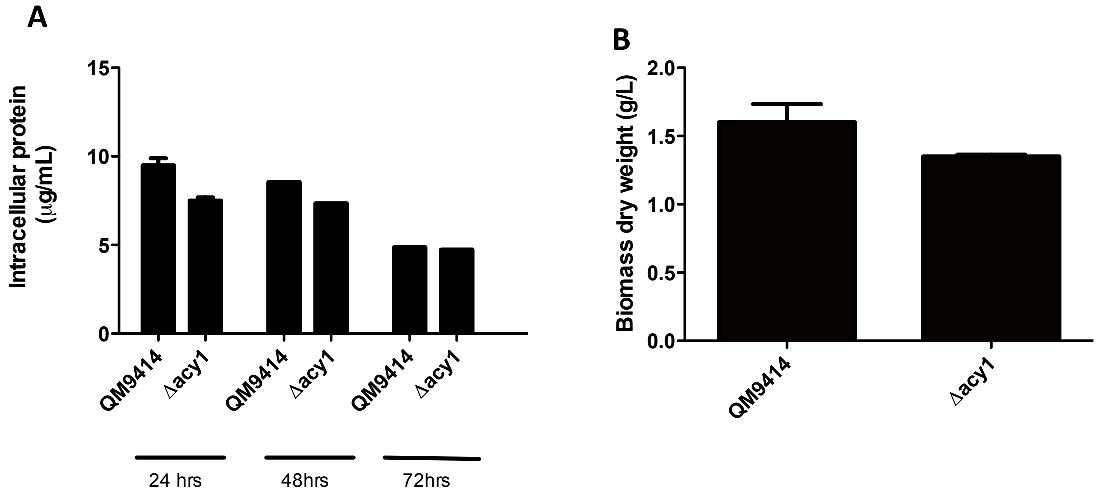

Additional file 1. Growth profiles of *T. reesei*, QM9414 and Δ*acy1* in cellulose (A) and glycerol (B) as carbon source. Error bars are represented from three biological replicates. . No significant difference was showed in growth between Δacy1 mutant strain and the parental QM9414
